# A core outcome set for neonatal abstinence syndrome: study protocol for a systematic review, parent interviews and a Delphi survey

**DOI:** 10.1186/s13063-016-1666-9

**Published:** 2016-11-08

**Authors:** Lauren E. Kelly, Lauren M. Jansson, Wendy Moulsdale, Jodi Pereira, Sarah Simpson, Astrid Guttman, Karel Allegaert, Lisa Askie, Henry Roukema, Thierry Lacaze, Jonathan M. Davis, Loretta Finnegan, Paula Williamson, Martin Offringa

**Affiliations:** 1 Child Health and Evaluative Sciences, the Hospital for Sick Children, Toronto, ON Canada; 2 Johns Hopkins University School of Medicine, Baltimore, MD USA; 3 Neonatal Intensive Care Unit, Aubrey and Marla Dan Program for High Risk Mothers and Babies, Sunnybrook Health Sciences Centre, Toronto, ON Canada; 4 Women’s and Infant’s Program, St. Joseph’s Healthcare, Hamilton, ON Canada; 5 Department of Pediatrics, the Hospital for Sick Children, Toronto, ON Canada; 6 Institute for Clinical Evaluative Sciences, Toronto, ON Canada; 7 Intensive Care and Department of Surgery, Erasmus MC-Sophia Children’s Hospital, Rotterdam, The Netherlands; 8 Department of Development and Regeneration, KU Leuven, Leuven, Belgium; 9 NHMRC Clinical Trials Centre, University of Sydney, Sydney, NSW Australia; 10 Faculty of Medicine and Dentistry, Department of Paediatrics, Western University, London, ON Canada; 11 Department of Pediatrics, Alberta Health Services and the Cumming School of Medicine, University of Calgary, Calgary, AB Canada; 12 Department of Pediatrics, The Floating Hospital for Children at Tufts Medical Center and the Tufts Clinical and Translational Science Institute, Boston, MA USA; 13 College on Problems of Drug Dependence, Philadelphia, PA USA; 14 Department of Biostatistics, University of Liverpool, Liverpool, England; 15 Faculty of Medicine, Department of Pediatrics, University of Toronto, Toronto, ON Canada

## Abstract

**Background:**

The prevalence of neonatal abstinence syndrome (NAS) is increasing globally resulting in an increased incidence of adverse neonatal outcomes and health system costs. Evidence regarding the effectiveness of NAS prevention and management strategies is very weak and further research initiatives are critically needed to support meta-analysis and clinical practice guidelines. In NAS research, the choice of outcomes and the use of valid, responsive and feasible measurement instruments are crucial. There is currently no consensus and evidence-based core outcome set (COS) for NAS.

**Methods/design:**

The development of the NAS-COS will include five stages led by an international Multidisciplinary Steering Committee: (1) qualitative interviews with parents/families and a systematic review (SR) to identify items for inclusion in a COS. The SR will also identify participants for the Delphi survey, (2) a three-round Delphi survey to gain expert opinion on the importance of health outcomes influencing NAS management decisions, (3), a consensus meeting to finalize the items and definitions with experts and COS users, (4) feasibility and pilot testing, development of the COS and explanatory document and (5) implementation planning.

**Discussion:**

Since standardized outcome measurement and reporting will improve NAS clinical research consistency, efficacy and impact, this COS will reflect the minimum set of health outcomes which should be measured in trials evaluating interventions for preventing or treating NAS.

**Electronic supplementary material:**

The online version of this article (doi:10.1186/s13063-016-1666-9) contains supplementary material, which is available to authorized users.

## Background

In the last decade, the “opioid epidemic” has seen a dramatic increase in the prevalence of opioid use [1, 2] driven primarily by prescription drugs and relatively inexpensive heroin. Not surprisingly, pregnant women have also been significantly impacted. In 2010, the use of prescription opioids during pregnancy was estimated at 14 % in the US [3]. Due to their liposolubility and low molecular weight, opioids pass through the placenta and are transmitted to the fetus. The rise in opioid use during pregnancy has caused a dramatic increase in the number of infants affected by NAS [4]. Postnatal opioid use in ventilated newborns and those undergoing surgical procedures further contribute to the burden of NAS. Nonopioid substances, including antidepressants, sedative-hypnotics, alcohol and tobacco, can potentiate the NAS development resulting in more severe expression [5–7]. Recent reports on NAS have declared a global epidemic, with prevalence ranging from 2 to 6 per 1000 live births [10].

NAS is a multisystem disorder characterized by disturbances in the central and autonomic nervous systems, the gastrointestinal tract and the respiratory system. If left untreated, NAS can cause death resulting from seizures, respiratory instability and/or fluid loss and may result in long-term visual or auditory impairments [8, 9]. The impact on the health system is considerable. The Canadian Institute for Health Information reported that in Ontario, newborns with NAS used an average of 23.4 hospital beds per day, up from 5.6 beds per day in 2003–2004. North American centers report consumption up to 45 % of neonatal intensive care unit (NICU) days for babies with NAS [10]. The true number of opioid exposures during pregnancy is difficult to capture as not all babies exposed will develop NAS and not all women are screened for opioid use in pregnancy, nor do they readily disclose this information [11]. Most important, while comprehensive global efforts are underway to address the opioid epidemic in adults, the care for newborns with NAS urgently needs attention [12].

There is no clinical best practice for the prevention or treatment of NAS. Reported management of NAS is variable and primarily based on eminence (experience)-based practices [12]. The efficacy of NAS management has been linked to several clinical and genetic factors; however, the evidence is very limited in view of the scope of the problem. In 2013, the WHO graded all available evidence on managing infants withdrawing from substance exposure in utero as “very low.” In October 2015, the US government passed the “Protecting Our Infants Act” (S.799) [13] committing resources to address research gaps, review existing practices and create a formal NAS surveillance program. In Canada and around the world, variability in measurement and reporting of NAS outcomes presents great challenges for health care providers, social workers, researchers, policy-makers and the community. The lack of harmonized, evidence- and consensus-based NAS health outcomes impedes progress in NAS clinical care and research. In the absence of harmonized outcome selection and reporting, site performance and research studies cannot be compared, contrasted nor combined, leading to inefficient research efforts [14]. There is an acute need for a *core outcome set* (COS) which is a minimum set of outcomes to be collected in clinical care and research in this disease area.

### Aim

The aim of this study is to develop a consensus-based COS for NAS to be used in clinical practice and research.

### Objectives

1. Synthesize evidence on maternal, neonatal and health care resource utilization outcomes into a comprehensive list of candidate outcomes for the COS
2. Interpret and prioritize NAS outcomes identified in the systematic review (SR) using parent interviews, a Delphi survey and a consensus meeting to develop a COS
3. Disseminate the NAS-COS to key stakeholders globally

## Methods

**Fig. 1:**
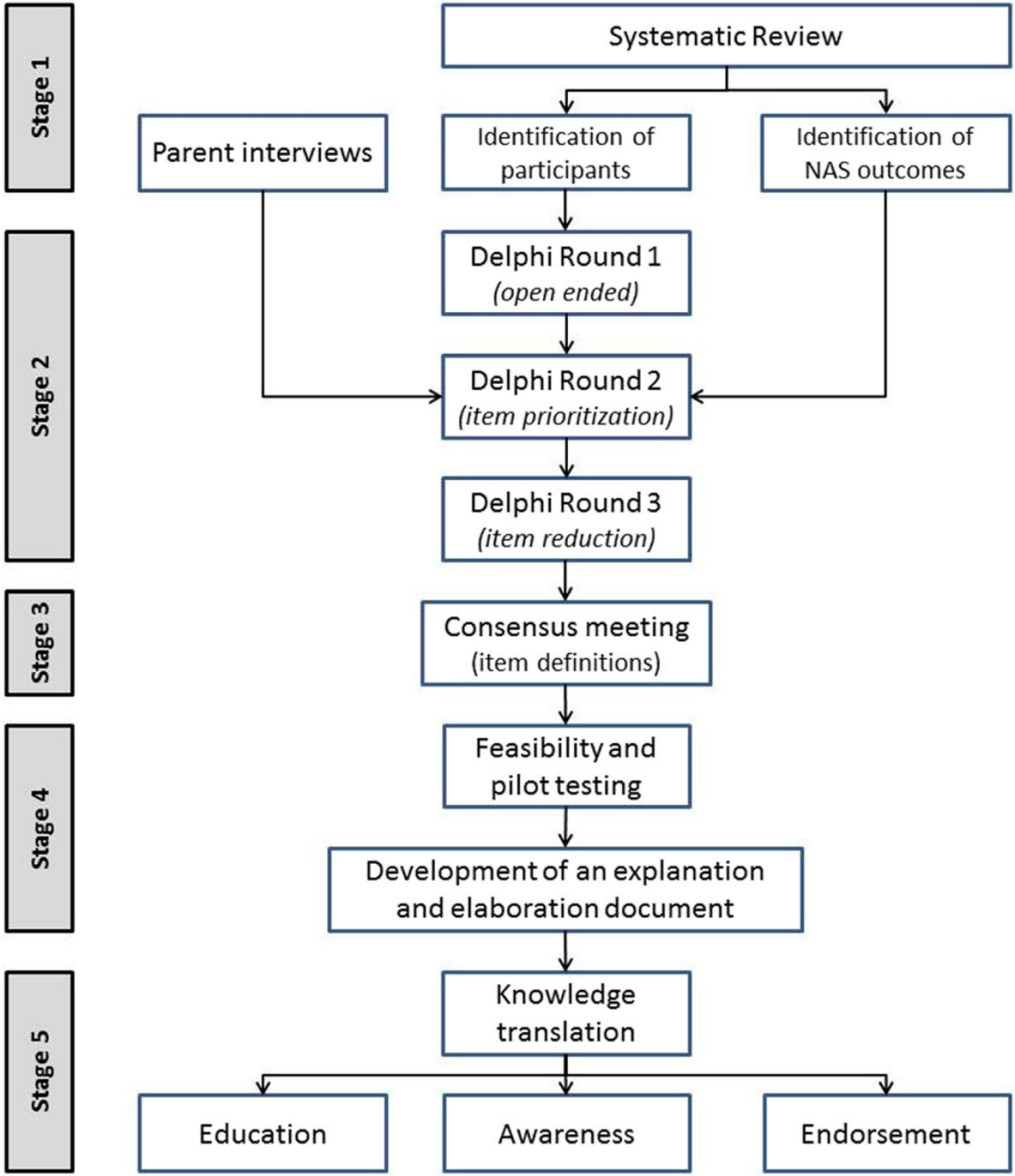
Project flow for the development of a core outcome set (COS) for neonatal abstinence syndrome NAS. NAS-COS development will include separate considerations for NAS resulting from (1) antennal opioid exposure (in utero) and (2) postnatal (iatrogenic) opioid use

This initiative has been registered with COMET (Core Outcome Measures in Effectiveness Trials) and PROSPERO (International Prospective Register of Systematic Reviews). We will use published recommendations for the development of a specific NAS-COS [15, 16], which in our case will include five stages as outlined in Fig. 1: (1) a SR and parent interviews to identify items for inclusion in a COS, (2) a three-round Delphi survey to gain expert opinion on the importance of health outcomes on influencing NAS management, (3) a consensus meeting to finalize the items and definitions, (4) pilot testing and the development of the COS explanatory document and (5) knowledge translation. Each of the following five stages (Fig. 1) is described in detail below.

### Stage 1

#### Systematic review

In 2010 Cochrane published two SRs assessing the effectiveness and safety of using an opiate compared to a sedative or nonpharmacological treatment for treatment of NAS [17, 18]. These reviews, which focus on management effectiveness in randomized or quasi-randomized trials, are currently in the process of being updated. We will identify outcomes reported in all NAS clinical research, including observational studies and clinical trials. To foster transparency and promote collaboration, the SR has been prospectively registered with PROSPERO. A comprehensive, electronic search was designed by a research librarian at the Hospital for Sick Children. All available English abstracts will be screened by two independent reviewers. Maternal and neonatal health outcomes will be extracted from full-text articles.

#### Types of studies, interventions and participants

Studies including SRs, clinical practice guidelines, randomized controlled trials, case-controlled trials, uncontrolled trials, case reports and observational cohort studies in NAS will be included. Reports of all interventions used to manage NAS will be analyzed. Although opioid-related NAS produces dramatic effects in neonates, other substances, including antidepressants, sedative-hypnotics, alcohol and tobacco, can contribute to the severity of NAS [5, 7]. For the purpose of this review, studies including neonates exposed to opioids (including methadone, oxycodone and other illicit and prescription opioids) in utero or postnatally who are diagnosed with NAS will be included. Separate consideration will be given to (1) in utero exposure and (2) postnatal exposure as these are often very different neonatal populations. Studies that do not describe NAS health outcomes or studies in which the full-text is not available in English will be excluded.

#### Search methods for identification of studies

The search strategy was developed with a librarian at the Hospital for Sick Children (Additional file 1). This search will be applied to the Web of Science, CINAHL, Cochrane Central, EMBASE and MEDLINE and reported according to the Preferred Reporting Items for Systematic Reviews and Meta-Analyses (PRISMA) guideline [19].

#### Eligibility of studies

Two independent reviewers will screen the abstracts resulting from all search strategies in EndNote X6. For all relevant studies, full-text articles will be obtained. Any disagreement in study eligibility criteria will be resolved by the principal investigator (PI). Studies will be excluded if they do not describe NAS health outcomes or if the full-text is not available in a language mastered by our team (English, French, Spanish or Dutch). Opioid-related NAS is the most common and produces the most dramatic withdrawal effects in neonates. Other substances including antidepressants, sedative-hypnotics, alcohol and tobacco can contribute to the severity and onset of the NAS symptoms [5, 7]. Only infants with a NAS diagnosis following opioid exposure in utero or postnatally will be included regardless of concomitant substance exposure.

#### Assessment of methodological quality

For each study that is included, the methodological quality of the reported outcomes will be assessed. As there is no synthesis of data for the reported health outcomes planned, the overall methodological quality of the study will not be evaluated. We will use six questions [20] to assess the quality of outcome reporting:

1. Is the primary outcome clearly stated?
2. Is the primary outcome clearly defined so that another researcher would be able to reproduce its measurement? (e.g. time points, person measuring outcome, measurement tools, location of outcome measurement)
3. Are secondary outcomes clearly stated?
4. Are the secondary outcomes clearly defined?
5. Do the authors explain the use of the outcomes they have selected?
6. Where applicable assess measurement tools. Were methods used to enhance the quality of outcome measurement? (e.g. training)

#### Data extraction, analysis and presentation

Data will be extracted independently by two reviewers. Discrepancies between data collection will be resolved by a senior reviewer. From each included study the following data will be extracted where available: journal of publication, authors, author affiliation/contact details, year of publication, study design, population (antenatal or postnatal exposure), type of exposure including duration of exposure, maternal dose (if in utero exposure), neonatal dose (if postnatal exposure), timing and dose at onset, inclusion and exclusion criteria, NAS intervention type, control arm, the number of patients included in the study, and inclusion and exclusion criteria. These data will be presented in a descriptive table and all reported outcome measures will be documented and the quality of outcome reporting for each study will be assessed. Of the outcomes selected for the COS, the validity of measurement instruments will be assessed using the most recent COSMIN list [21]. No formal sample size has been calculated for the Delphi process. Approximately 150 participants from around the world, identified through the SR and Steering Committee will be invited to participate.

#### Data interpretation, validation and prioritization

In order to contextualize the outcomes identified by the SR, we will engage with health care providers and parents with real-world NAS experience. In order to ensure that outcomes identified during the SR are relevant and meaningful in both a research and clinical care setting, we will integrate findings from parent interviews, the Delphi survey and the consensus meeting with key stakeholders into the final COS.

#### Identification of outcomes important to parents and families

It is important that the outcomes collected in both research and routine care are meaningful to the patients and families receiving care. As the patients in this case are neonates, parents and families will be provided with the opportunity to contribute to selecting appropriate outcomes for infants with NAS. As the COS will include both maternal and neonatal health outcomes the involvement of mothers of newborns with NAS is critical. A purposive sample, minimum of four caregivers (parents, foster parents, or legal guardians) will be selected at the coauthors’ substance abuse treatment centers. Parents of infants displaying symptoms of NAS will be asked if they would like to participate and offered a $20 CAD gift card to a local eatery. The aim will be to select a representative sample accounting for ethnic, socioeconomic and demographic distribution which includes both birth and foster parents/caregivers. Parents/caregivers will complete qualitative interviews via an open-ended approach where they will be asked to describe their experiences with their infant’s NAS treatment and the impact on their families. These interviews will be completed at discharge so that caregivers do not feel as though their decision to participate or their responses will affect their infants care. The goal of these interviews will be to gather evidence of important issues raised by parents, caregivers and families. These semistructured, open-ended interviews will include a topic guide to ensure that all identified health outcomes are discussed and the importance of each outcome is assessed. Ethics board approval for the qualitative interviews will be obtained prospectively and informed consent will be obtained. All interviews will be recorded and transcribed verbatim. Qualitative analysis software will be used to identify themes in the recordings and to identify outcome domains important to parents and families. These outcome measures will be identified independently from the SR.

### Stage 2

#### Identification of outcomes important to health care providers

A three-round Delphi approach has been selected to investigate the importance of NAS outcomes to neonatologists, obstetricians, midwives, substance abuse treatment providers, primary care physicians, nurses, nurse practitioners and social workers. The Delphi will be conducted using secure Delphi Manager® software hosted at the University of Liverpool and, with the exception of the open-ended Delphi round 1, will be completed using SurveyMonkey. The Delphi survey will be completed anonymously in order to account for all participants’ opinions, to avoid influence from other participants, and to maintain confidentiality. Only the study team will have access to the participant contact information.

#### Participants

A wide variety of stakeholders, including all corresponding authors of studies included in the SR, will be invited to participate via e-mail. The e-mail will outline the project including the timelines. Additional international experts in neonatology, nursing, obstetrics, social work and opioid exposure in pregnancy may be consulted at the discretion of the Steering Committee. For applicability, the NAS-COS aim to be relevant for international settings across a variety of domains. The number of participants who are invited will be recorded along with demographic information including, years of experience, country of practice and qualifications. All corresponding authors identified in the SR and knowledge users identified by the Steering Committee will be invited to participate via e-mail. For applicability, the NAS-COS participants will include future potential NAS-COS users; we expect over 150 individuals to be invited. Response and attrition rates will be reported. Although development of COS generally includes patients in the Delphi survey [15], the authors felt that engagement through interviews was better suited to interpret outcomes from a family perspective given the nature of the patients (newborns). Participants will include NAS-COS users. Parent and family involvement will be sought during focus groups.

#### Delphi round 1

Participants will be assigned a unique identification code and will not be able to see the responses or identification of any other participants. Individuals will be invited via e-mail to participate in round 1 of the Delphi survey where they will consent to being contacted for rounds 2 and 3. The number of respondents who complete all rounds will be recorded with response and attrition rates being calculated and reported. Participants will be provided with study information, including institutional approvals and contact information for questions. Prior to distribution, the Delphi survey will be approved by the Quality Improvement Team at the Hospital for Sick Children. Previous COS developers have confirmed that the National Research Ethics Committee does not require Research Ethics Board approval for Delphi surveys involving clinicians [20].

The first question will ask if participants are interested in the development of a COS for NAS. If they agree, the remaining survey questions will be populated. The first round of the Delphi survey will collect demographic information on the participants and will present the study rationale. Unidentified demographic information will include: number of years of practice, location of practice, and clinical role (e.g. nurse, neonatologist, obstetrician, researcher, pharmacist, etc.). Round 1 will be open for 4 weeks and reminder e-mails will be sent weekly to those invited. The survey will be presented in an online format and will be piloted within the Steering Committee prior to distribution to assess clarity prior to distribution. To ensure outcomes identified by the SR are relevant to a wide range of NAS health care providers, round 1 will be an open-ended question: “Thinking about your clinical and research practice, please list up to 10 outcomes that you feel are important to inform NAS diagnosis and management.” For each health outcome, participants will be provided with a text box to describe how they would measure this outcome. Any type of outcome (laboratory test, questionnaire, etc.) can be included [22].

#### Delphi round 2 (outcome prioritization)

Round 2 of the Delphi will also be provided in an electronic format using Delphi Manager®. To ensure that a comprehensive set of NAS health outcomes is generated, health care providers will be asked to prioritize outcomes collected from: (1) round 1 of the Delphi survey, (2) the SR (published literature), and (3) from parent interviews with each item including the source of information (experts, literature, families). Outcomes will be grouped under the appropriate domains, according to OMERACT domains (Death, Life Impact, Pathophysiological Manifestations and Resource Use) [23, 24] presented in alphabetical order to avoid the appearance of a hierarchy. Participants will be asked “What is the importance of this health outcome in influencing your treatment of NAS?” The outcome rating scale will range from 1 to 9,where 1 to 3 are labeled “Not important for inclusion in the NAS-COS,” 4 to 6 are labeled “Important but not critical for inclusion in the NAS-COS” and 7 to 9 are labeled “Critical for inclusion into the NAS-COS” [24]. Participants will also be asked how they would measure each presented outcome. This initiative has been registered with COMET and PROSPERO [25]. Following the importance rating of items identified from the SR, an option to add in additional health outcomes which they currently view as influencing care will be provided in an open-text format.

#### Delphi round 3 (item reduction)

Participants will be presented with the results (mean, median and range) for the top-scoring items from round 2. They will then be asked for each outcome if they feel that this outcome would influence their care and should be included on a NAS-COS where 0 = never, 1 = sometimes and 2 = always. To ensure that each outcome is completely reported, outcome items will also include data collected regarding measurement tools. For example, with regarding the item #1 Neonatal Abstinence Severity Score, your preferred measurement tool is (a) Modified Finnegan score, (b) Lipsitz Neonatal Drug-withdrawal scoring system, (c) Neonatal Withdrawal Inventory, (d) Neonatal Narcotic Withdrawal Index and (e) any other scoring systems identified during the SR. For each scale used in practice, participants will be asked to report (1) how frequently infants are scored; (2) what is the cut-off points for initiating pharmacologic management? (3) who is responsible for completing the scoring; and (4) how long the neonates remain in hospital for observation.

### Stage 3

#### Consensus meeting

Through purposive sampling, approximately 20 participants from diverse stakeholder groups, including physicians, nurses, nurse practitioners, midwives, social workers, researchers and parent representatives, will be invited to participate in a face-to-face meeting with the Steering Committee. Journal editors, industry representatives and regulators will also be invited to attend. The purpose of the consensus meeting is to finalize the COS and to define each outcome and measurement tool. The consensus meeting will be conducted via a Nominal Group Process [26]. The results from the Delphi survey will be presented as well as qualitative data from the parent interviews. Similar to other COS development protocols [19], consensus will be determined if 70 % or more participants scored the item with an importance of 7 to 9 *and* less than 15 % of participants scored the item as 1 to 3. All included and excluded items will be reviewed during this time.

### Stage 4

#### Pilot testing

The Steering Committee and those invited to the consensus meeting will be invited to pilot test the COS. Experienced COS developers will be identified through COMET and consulted on the overall NAS-COS. The International Neonatal Consortium (INC) is a public-private partnership involving global regulators, members of the academic community, families, foundations and industry representatives united in the goal of fostering neonatal clinical research. Members of INC will be invited to provide feedback on the overall relevance of the NAS-COS for clinical and research practice.

#### Development of the COS and explanatory document

The COS reporting guideline will include all health outcomes and measurement procedures as determined by the parent interviews, Delphi rounds and consensus meeting. The explanatory document will contain all of the background, rationale and justification for each health outcome and will be developed concurrently with the COS reporting guideline. All Delphi survey participants will be provided with the final COS document and given the opportunity to provide feedback on content, format, and usefulness. Details regarding the reported measurement of each identified NAS health outcome will also be included to foster harmonization of data collection. Examples of good reporting will be provided in the explanation document. The first drafts will be developed by the project leaders and distributed to the Steering Committee for comment and approval.

### Stage 5

#### Knowledge translation

A central priority of this research is to increase awareness regarding the importance of COS in the field of NAS research and treatment. Active involvement of partners will be achieved by bringing representatives together from diverse international stakeholder groups in the selection and prioritization of NAS health outcomes and subsequent development of the COS. Stakeholders will remain engaged throughout the evaluation and implementation process, and be provided with an active role in the strategic planning on actions to amplify the impact of the NAS-COS.

Our audience includes:

1. Researchers and health care providers – to use the NAS-COS in future clinical research (e.g. Canadian Association of Pediatric Health Centers, American Academy of Pediatrics, Canadian Pediatric Society, The Society of Obstetricians and Gynecologists of Canada)
2. Systematic reviewers – to use the NAS-COS to identify important outcomes and promote the use of NAS-COS in future research (e.g. Cochrane Child Health, Cochrane Neonatology)
3. Funding agencies and journals – to encourage the use of NAS-COS in future research (e.g. CIHR, NIH, *JAMA Pediatrics*, *Pediatrics*)
4. Health care system – to use NAS-COS for routine monitoring and plan quality improvement activities (e.g. European Medicines Agency, Food and Drug Administration, Health Canada)

Our integrated knowledge translation plan encompasses education, dissemination, and endorsement by various key stakeholders as summarized in Table 1. Our involvement of all corresponding authors and a wide variety of knowledge users (neonatology, health policy, perinatal addiction, pharmacology, midwifery, nursing and social work) in the development of the COS will maximize implementation by NAS research teams and clinicians around the world. Additional funding will be sought for a formal prospective evaluation of the NAS-COS including a survey of users citing the use of the NAS-COS 5 years post publication. This evaluation will include a plan for quality improvement and examination of implementation barriers such as a lack of training or cost.

**Table 1:** Neonatal abstinence syndrome core outcome set (NAS-COS) knowledge translation plan

Education	• Our collaborators have committed to including the NAS-COS in the next edition of the Ontario NAS guidelines and the Canadian Pediatric Society NAS Practice Point
	• Methods for developing the NAS-COS will be reported in a separate publication which will be written according to the reporting guideline for studies developing COS
	• Registration and protocol publication will raise awareness of this COS development, encourage collaboration, and provide expert resources and increase uptake
	• Collaborative training initiatives within the Canadian Child Health Clinician Scientist Program and Women and Babies Clinical Educators Network
Dissemination	• The results from the systematic review (SR) will be published, focusing on the quality of outcome reporting and variability in outcome selection/measurement
	• The SR and COS findings will be disseminated through several large international organizations including: the European Society of Pediatric Research, the European Society of Developmental Pharmacology, International Neonatal Consortium, Perinatal Society of Australia, American Academy of Pediatrics, Canadian Neonatal Nurses Association, American Pediatric Society, Neonatal Advisory Committee at the Food and Drug Administration, CPS Fetus and Newborn Committee, and the Canadian Neonatal Network (CNN)
	• The COS will be presented at international conferences to foster awareness globally. Funds are requested to present this work at the Pediatric Academic Societies, American Academy of Pediatrics, and the European Society for Pediatric Research annual meetings
Endorsement	• The Steering Committee will use the NAS-COS to inform the design of our own planned clinical research in this area
	• Update the Canadian BORN and CNN databases
	• Published in neonatology journals
	• Prospective engagement with Core Outcome Measures in Effectiveness Trials (COMET)

The methods for developing the NAS-COS are being drafted for publication and will be written according to the reporting guideline for studies developing a COS [4]. Registration and protocol publication will raise awareness of the COS development, encourage collaboration, provide expert resources and increase uptake. The NAS-COS will be published in a journal with a neonatology focus and CROWN (CoRe Outcomes in WomeN’s health) will be consulted. Journals which publish neonatology research will be contacted to support NAS-COS implementation. Additional journals may be identified through the SR. To foster transparency, the authors plan to publish the results from the SR in the explanatory document that focuses on the quality of outcome reporting and variability in NAS. These data will be presented at international conferences to foster dissemination globally.

Neonatal specialty groups, identified through INC will be presented with the COS and asked to disseminate to their members. As there are relatively few investigators conducting NAS research, involving all corresponding authors in the design of the COS will maximize implementation by NAS research teams around the world. A formal evaluation of the COS will include a survey of users citing the use of the NAS-COS 5 years post publication. This evaluation will include a plan for quality improvement and evaluate implementation barriers such as a lack of training or cost.

## Discussion

According to the Canadian Pediatric Society (CPS) there is no best practice for managing newborns with NAS in Canada or in other parts of the world. As the health care costs associated with NAS management are six times the costs associated with non-NAS births [27], the increasing number of NAS cases, locally occupying more than 20 % of NICU days, is a worrisome resource burden. Research done to establish treatment effectiveness must select and report qualified, valid and feasible health outcomes. In order to accelerate the discovery of beneficial treatment strategies, reduce research waste and optimize resource allocation, high-quality effectiveness evidence must be generated and synthesized. Efforts to improve the quantity and quality of NAS research have been highlighted by the call to action of the US Congress and NIH.

There is currently no published COS for NAS. This paper describes the development of evidence and consensus-based COS aims to improve the comparison of future studies, improve research quality and harmonize outcome selection/measurement. This review will provide a current and transparent assessment of the NAS health outcomes that will inform clinical practice and the design of large multicenter comparative effectiveness trials. The NAS-COS is a first step in facilitating high-quality evidence that will ease the comparability between studies and foster meta-analysis. The NAS-COS will incorporate key stakeholder groups, including parents and families to develop a meaningful and feasible set of health outcomes. Through engaging health care providers and families with real-world NAS experience to interpret and prioritize the NAS outcomes, we will generate a meaningful and relevant COS to inform the design of large, multicenter comparative effectiveness trials, prospective SRs, and meta-analyses with the goal of markedly improving care for this highly vulnerable population.

## Additional file

Additional file 1: Search strategy. (PDF 176 kb)
