# Supplementary material for: A core outcome set for neonatal abstinence syndrome: study protocol for a systematic review, parent interviews and a Delphi survey
Source: Trials. 2016 Nov 8;17:536. doi: 10.1186/s13063-016-1666-9 (PMC5100193; doi:10.1186/s13063-016-1666-9)
Supplement: Additional file 1: — Search strategy. (PDF 176 kb) [file 13063_2016_1666_MOESM1_ESM.pdf]

Appendix 1: Search strategy for systematic review

Database: Ovid MEDLINE(R) 1946 to Present with Daily Update, Ovid MEDLINE(R) In-Process & Other Non-Indexed Citations <December 22, 2015>
Created by Alanna Marson, Reference and Instruction Librarian, Library and Archives Services, the Hospital for Sick Children

Search Strategy:
----
1 Neonatal Abstinence Syndrome/ (932)
2 (neonat* adj3 Abstinence).tw,kf. (514)
3 ((withdraw* or abstinence) adj3 (substance* or symptom* or syndrome* or drug* or opioid* or opiate*)).tw,kf. (19016)
4 (passive adj3 addiction*).tw,kf. (15)
5 Substance Withdrawal Syndrome/ (20258)
6 3 or 4 or 5 (32102)
7 (infant* or infancy or newborn* or new-born* or baby* or babies or neonat* or toddler*).mp. (1314214)
8 6 and 7 (1861)
9 1 or 2 or 8 (2326)

4 duplicates removed.
2322 total from Medline.
